# Disrupted tenogenesis in masseter as a potential cause of micrognathia

**DOI:** 10.1038/s41368-022-00196-y

**Published:** 2022-10-18

**Authors:** Chao Liu, Nan Zhou, Nan Li, Tian Xu, Xiaoyan Chen, Hailing Zhou, Ailun Xie, Han Liu, Lei Zhu, Songlin Wang, Jing Xiao

**Affiliations:** 1 Department of Oral Pathology, Dalian Medical University School of Stomatology, Dalian, China; 2 Academician Laboratory of Immunology and Oral Development & Regeneration, Dalian Medical University, Dalian, China; 3 Beijing Laboratory of Oral Health, Capital Medical University, Beijing, China

**Keywords:** Bone development, Mandibular muscles

## Abstract

Micrognathia is a severe craniofacial deformity affecting appearance and survival. Previous studies revealed that multiple factors involved in the osteogenesis of mandibular bone have contributed to micrognathia, but concerned little on factors other than osteogenesis. In the current study, we found that ectopic activation of *Fgf8* by *Osr2-cre* in the presumptive mesenchyme for masseter tendon in mice led to micrognathia, masseter regression, and the disrupted patterning and differentiation of masseter tendon. Since *Myf5-cre;Rosa26R-Fgf8* mice exhibited the normal masseter and mandibular bone, the possibility that the micrognathia and masseter regression resulted directly from the over-expressed *Fgf8* was excluded. Further investigation disclosed that a series of chondrogenic markers were ectopically activated in the developing *Osr2-cre;Rosa26R-Fgf8* masseter tendon, while the mechanical sensing in the masseter and mandibular bone was obviously reduced. Thus, it suggested that the micrognathia in *Osr2-cre;Rosa26R-Fgf8* mice resulted secondarily from the reduced mechanical force transmitted to mandibular bone. Consistently, when tenogenic or myogenic components were deleted from the developing mandibles, both the micrognathia and masseter degeneration took place with the decreased mechanical sensing in mandibular bone, which verified that the loss of mechanical force transmitted by masseter tendon could result in micrognathia. Furthermore, it appeared that the micrognathia resulting from the disrupted tenogenesis was attributed to the impaired osteogenic specification, instead of the differentiation in the periosteal progenitors. Our findings disclose a novel mechanism for mandibular morphogenesis, and shed light on the prevention and treatment for micrognathia.

## Introduction

Micrognathia is a severe congenital deformity characterized by the miniature or shortened mandible.^1^ Clinically, human newborns suffering from micrognathia are usually prone to dyspnea or feeding intolerance because the tongue pushed up- and back-ward by the reduced oral cavity (glossoptosis/retroglossia) obstructs pharynx.^2^ More severely, the glossoptosis/retroglossia will lead to Pierre-Robin Sequence (PRS) in the instance that the undescended tongue blocks the elevation of palatal shelves, which results in clefting of the palate.^3,4^ The incidence of micrognathia or PRS was reported arranging from the one out of 3 000–5 600 births, to one in every 8 500–14 000 newborns.^5,6^ Approximately one third of micrognathia or PRS cases are reported in a variety of syndromes, including Stickler syndrome, 22q11 deletion syndrome, Central Hypoventilation Syndrome, Treacher Collins Syndrome, etc.^7^ According to the studies on these syndromes, a number of gene mutations, mainly involved in the development of neural crest cells, are implicated as the causative factors of micrognathia or PRS.^8–12^ However, over two thirds of micrognathia or PRS cases are sporadic or non-syndromic with still unknown reasons.^7^

The mandibular skeleton is predominantly originated from the neural crest-derived mesenchyme which delaminate from the boundary between surface and neural ectoderm at the hindbrain.^13^ The delaminated neural crest cells immigrate into mandibular process where they undergo specification and morphogenesis, and eventually differentiate into the mature mandible skeleton via intramembranous ossification.^14^ Currently, *Distal-less homeobox5/6* (*Dlx5/6*), *Endothelin-1* (*Edn1*)/ *Endothelin A Receptor* (*Ednra*) and *Hand 2* are regarded as the determinants of mandibular specification. All of *Dlx5*^*−/−*^*;Dlx6*^*−/−*^, *Edn1*^*−/−*^ and *Ednra*^*−/−*^ mice transform their mandibular arches into maxillary-like structures.^15–17^ On the other hand, mice over-expressing *Ednra* or *Heart and neural crest derivatives expressed 2* (*Hand2*) even convert the maxillary processes into mandibular-like structures.^18,19^ In addition, these genes are also involved in mandibular morphogenesis. The haploinsufficiency of the single or both *Dlx5* and *Dlx6* genes exhibited a dosage-dependent effect on mandible size, namely, the more *Dlx5* and/or *Dlx6* alleles were inactivated, the shorter mandible became.^17,20,21^ Similarly, conditional inactivation of *Edn1* in the ectoderm and/or mesoderm, as well as conventional abrogation of *Hand2* in mice, result in the hypoplastic mandibles.^22–24^ In addition to the mandibular specification and morphogenesis, the chondrogenesis and osteogenesis of mandibular skeleton are also critical for the normal mandible size. Tissue-specific inactivation of *SRY-box transcription factor 9* (*Sox9*) or *Connective Tissue Growth Factor* (*Ctgf*) in neural crest leads to micrognathia in mice, indicating that the fate decision and proliferation in Meckel’s cartilage are essential for the normal size of mandibular bones.^25–27^ Moreover, several studies found that the interrupted non-canonical Transformation Growth Factor β/Bone Morphogenic Protein (TGFβ/BMP) signaling could lead to micrognathia and PRS by impairing the osteogenic differentiation of mandibular mesenchyme.^28–30^ Taken together, it is suggested that any disruption in the specification, morphogenesis and differentiation of mandibular mesenchyme can shorten the mandible.

Most studies on micrognathia and PRS focused on the disorders in the mandibular bone.^31^ However, the micrognathia in the *Myogenic Differentiation* (*MyoD*) and *Myogenic Factor 5* (*Myf5*) double knock-out mice indicated that the failure of myogenesis or the loss of muscle contraction also results in a shortened mandibular bone,^32,33^ which verifies the notion that the coordination between skeletogeneis and myogenesis is indispensable for normal development and postnatal function of musculoskeletal system.^34–37^ Musculoskeletal system is composed of bones, tendons, and muscles. Previous study demonstrated that tenogenesis is involved in long bone morphogenesis not only by transmitting the force generated by muscle contraction to bone, but also by secreting BMP4 and Fibroblast Growth Factor 4 (FGF4) to shape the secondary structures of long bones.^38^ Therefore, whether craniofacial tendons play the similar roles in mandible morphogenesis as craniofacial muscles do requires to be elucidated.

Both craniofaical bones and tendons are derived from the craniofacial neural crest. Although it is still unknown how craniofacial neural crest cells are specialized into osteogenic and tenogenic fates, Mohawk Homeobox (Mkx), Early Growth Response 1 (Egr1) and Scleraxis (Scx) are found as the key transcription factors to tenogenic differentiation, while Sox9, Runt-related transcription factor 2 (Runx2) and Osterix (Osx) to osteogenic specification.^39^ Previous studies exploiting avian limb tendons displayed the activation of *Fgf4*, *Fgf8*, *Fgfr1*, as well as *Sprouty1* and *2* (the intracellular inhibitors to FGF signaling) in the developing tendons,^40,41^ implicating a role of FGF signaling during tenogenesis However, it is FGF4, instead of FGF8, that promotes tendon development and *Fgf8* transcripts are not detected in the developing mouse limb tendons.^41^ Thus, the role of FGF8 in mammalian tendon development requires further exploration. Our previous and recent studies showed that FGF8 dramatically suppresses the differentiation of neural crest-derived mesenchymal cells by sustaining the stem cell status.^42^ During palatogenesis, FGF8 could convert the osteogenic fate of palatal mesenchyme into chondrogenic fate.^43,44^ Therefore, to examine whether FGF8 impacts the mammalian tendon development, and whether the compromised craniofacial tenogeneis contributes to micrognathia, we activated a conditional *FGF8* transgene in the *Rosa26* locus by the *Odd-Skipped Related 2-cre* (*Osr2-cre*) knock-in allele in the progenitors of masseter tendon.

## Results

### *Osr2-cre* is activated in the developing masseter tendon but excluded from the masseter and mandibular skeleton

**Fig. 1:**
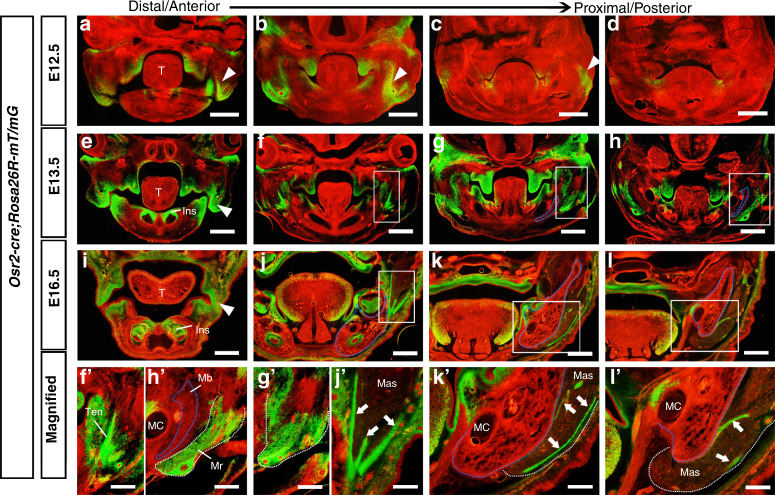
*Osr2-cre; Rosa26R-mT/mG* mice showed the *Osr2-cre* pattern in the developing craniofacial region. **a**–**d** Cryostat sections of the E12.5 *Osr2-cre; Rosa26R-mT/mG* facial region. The white arrowheads in **a**–**c** indicated Cre activity (green fluorescence) in the presumptive masseter tendon. **e**–**h** Cryostat sections of the E13.5 *Osr2-cre; Rosa26R-mT/mG* facial region. The areas in the boxes of **f**–**h** were magnified in **f’**, **g’** and **h’**, respectively. The white arrowheads in **e** indicated the Cre activity (green fluorescence) in the presumptive masseter tendon. **i**–**l** Cryostat sections of the E16.5 *Osr2-cre; Rosa26R-mT/mG* facial region. The white arrowheads in **i** indicated the Cre activity (green fluorescence) in the presumptive masseter tendon. The areas in the boxes of **j**–**l** were magnified in **j’**, **k’** and **l’**, respectively. The white arrows in **j’**, **k’** and **l’** pointed to the forming tendons of masseter or mylohyoideus. The blue dotted lines circled mandibular bones, while the white dotted lines delineated masseter tendons; T tongue, Ins incisor, MC Meckel’s cartilage, Mb mandibular bone, Mr masseter region, Mas masseter myofibers, Mhs mylohyoideus myofibers, Ten tendon. Scale bars: 200 μm

To address the *Osr2-cre* expression pattern during craniofacial development, *Osr2-cre;Rosa26R-mT/mG* mouse embryos were collected for cryostat sections. The E12.5 *Osr2-cre;Rosa26R-mT/mG* craniofacial cross sections showed that Cre activity was widely distributed in the anterior and middle palatal mesenchyme (Fig. 1a, b), but weakly or even absent in the posterior palatal mesenchyme (Fig. 1c, d). The E12.5 incisor mesenchyme (Fig. 1a) and the oral mesenchyme lateral to the tongue (Fig. 1b–d) also showed *Cre* activity. In addition, *Cre* expression was also activated in the lateral mesenchyme at the most anterior level of the mandibular and maxillary arches (Fig. 1a), which joined together and got the maximal domain at the middle level (Fig. 1b), but reduced in the posterior levels (Fig. 1c, d). In the E13.5 mandibular and maxillary arches, the *Cre*-expressing domains expanded throughout the palatal, incisor and oral mesenchyme (Fig. 1e–h). Interestingly, at this moment, the Cre activity in the mesenchyme connecting maxillary and mandibular arches was concentrated in the presumptive masseter tendons from anterior to posterior (Fig. 1f–h). Worthy of noticing, although detected in the masseter region, the Cre activity was found only in the tenogenic mesenchyme, as apposed of the myogenic and osteogenic compartments (Fig. 1f’, g’, h’). At E16.5, Cre activity further extended to molar mesenchyme and the peripheral mesenchyme of tongue (Fig. 1i–l). In the masseter area, Cre activity was confined to the deep masseter tendons and subcutaneous tissues at the middle level (Fig. 1j’), as well as to the superficial masseter tendons at the posterior levels (Fig. 1k’, l’). In contrast, the E16.5 masseter myofibers, Meckel’s cartilage, and mandibular bone were devoid of Cre activity.

### *Osr2-cre;Rosa26R-Fgf8* mice exhibit micrognathia

**Fig. 2:**
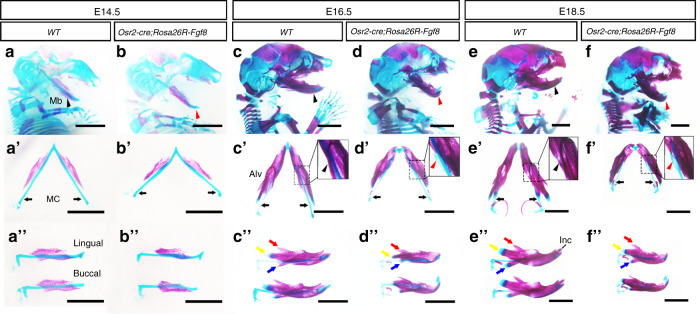
The mandibular skeleton in *Osr2-cre;Rosa26R-Fgf8* mice. **a**, **a’**, **a”**, **b**, **b’**, **b”** The lateral views of the bone and cartilage staining for the E14.5 WT (a) and *Osr2-cre;Rosa26R-Fgf8* craniofacial skeleton (**b**). The black arrowhead in **a** and the red arrowhead in **b** pointed to the mandibular skeleton. The ventral views of the E14.5 WT (**a’**) and *Osr2-cre;Rosa26R-Fgf8* mandibular skeleton (**b’**). The black arrows in **a’** and **b’** pointed to the terminus of Meckel’s cartilage. The lingual and buccal views of the E14.5 WT (**a”**) and *Osr2-cre;Rosa26R-Fgf8* mandibular skeleton (**b”**). **c**, **c’**, **c”**, **d**, **d’**, **d”** The lateral views of the bone and cartilage staining for the E16.5 WT (**c**) and *Osr2-cre;Rosa26R-Fgf8* craniofacial skeleton (**d**). The black arrowhead in **c** and the red arrowhead in **d** pointed to mandibular bone. The ventral views of the E14.5 WT (**c’**) and *Osr2-cre;Rosa26R-Fgf8* mandibular skeleton (**d’**). The black arrows in **c’** and **d’** pointed to Meckel’s cartilage. The dashed boxes in **c’** and **d’** were magnified in the solid boxes, in which the black arrowhead indicated the WT Meckel’s cartilage, while the red arrowhead pointed to the extra cartilage in *Osr2-cre;Rosa26R-Fgf8* Meckel’s cartilage. The lingual and buccal views of the E14.5 WT (**c”**) and *Osr2-cre;Rosa26R-Fgf8* mandibular skeleton (**d”**). The red, yellow and blue arrows in **c”** and **d”** delineated the coronoid, condylar and angular processes, respectively. **e**, **e’**, **e”**, **f**, **f’**, **f”** The lateral views of the bone and cartilage staining for the E18.5 WT (**e**) and *Osr2-cre;Rosa26R-Fgf8* craniofacial skeleton (**f**). The black arrowhead in **e** and the red arrowhead in **f** pointed to the mandibular skeleton. The ventral views of the E14.5 WT (**e’**) and *Osr2-cre;Rosa26R-Fgf8* mandibular skeleton (**f’**). The black arrows in **e’** and **f’** pointed to Meckel’s cartilage. The dashed boxes in **e’** and **f’** were magnified in the solid boxes, in which the black arrowhead indicated the degenerated Meckel’s cartilage in WT mandible, while the red arrowhead pointed to the extra and persistent Meckel’s cartilage in *Osr2-cre;Rosa26R-Fgf8* mandible. The lingual and buccal views of the E14.5 WT (**e”**) and *Osr2-cre;Rosa26R-Fgf8* mandibular skeleton (**f”**). The red, yellow and blue arrows in **e”** and **f”** delineated the coronoid, condylar and angular processes, respectively. Mb Mandible, MC Meckel’s cartilage, Inc incisor. Scale bars: 2 mm

Even Cre activity was excluded from the mandibular bone, the *Osr2-cre; Rosa26R-Fgf8* mandibles were noticeably shorter than WT controls from E14.5 (Fig. 2a, b; Supplementary Fig. 1). Interestingly, although the lengths of Meckel’s cartilages showed no significant difference between *Osr2-cre;Rosa26R-Fgf8* and WT mice (Supplementary Fig. 1), the distance between the terminus of the *Osr2-cre;Rosa26R-Fgf8* Meckel’s cartilage was obviously wider (Fig. 2a’, b’), which resulted in micrognathia by shortening the anterior-posterior length of the mandible. Moreover, the ossified bone of E14.5 *Osr2-cre;Rosa26R-Fgf8* mandible showed mildly shorter than the WT mandibular bone, implicating an impaired osteogenesis in the *Osr2-cre;Rosa26R-Fgf8* mandibular bone. The micrognathia in *Osr2-cre;Rosa26R-Fgf8* mice became evident at E16.5 (Fig. 2c, d; Supplementary Fig. 1), in which the Meckel’s cartilage not only was significantly shorter than WT control, but also displayed an extra cartilage (Fig. 2c’, d’; Supplementary Fig. 1). In addition, the ossified *Osr2-cre;Rosa26R-Fgf8* mandibular bone was also remarkably shorter with the reduced condylar, coronoid and angular processes (Fig. 2c”, d”). At E18.5, the micrognathia in *Osr2-cre; Rosa26R-Fgf8* mice became more severe (Fig. 2e, f), which was characterized by the obviously shorter mandibular bone, as well as the extra Meckel’s cartilage (Fig. 2e’, f’). In contrast to the secondary structures in the WT mandibular bone (Fig. 2e”), both the osteogenic and chondrogenic compartments of the E18.5 *Osr2-cre;Rosa26R-Fgf8* condylar, coronoid and angular processes were severely reduced (Fig. 2f”).

### Disrupted tenogenesis and regressed myofibers in *Osr2-cre;Rosa26R-Fgf8* masseter

**Fig. 3:**
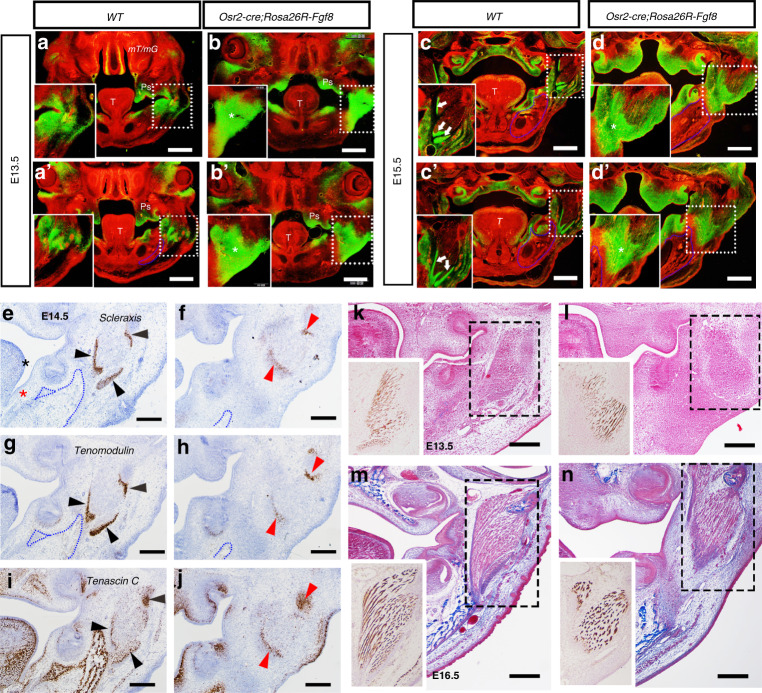
The tenogenesis and myogenesis in *Osr2-cre; Rosa26R-Fgf8* masseter. **a**, **a’** The cross views of E13.5 *Osr2-cre;Rosa26R-mT/mG* mice at the deep (**a**) and superficial (**a’**) masseter tendons. **b**, **b’** The cross views of E13.5 *Osr2-cre; Rosa26R-mT/mG; Rosa26R-Fgf8* mice at the deep (**b**) and superficial (**b’**) masseter tendons. The asterisks in **b** and **b’** delineated the tenogenic mesenchyme of masseters. **c**, **c’** The cross views of E15.5 *Osr2-cre;Rosa26R-mT/mG* mice at the deep (**c**) and superficial (**c’**) masseter tendons. The white arrows in **c** and **c’** delineated the masseter tendons. **d**, **d’** The cross views of E15.5 *Osr2-cre; Rosa26R-mT/mG; Rosa26R-Fgf8* mice at the deep (**d**) and superficial (**d’**) masseter tendons. The asterisks in **d** and **d’** delineated the tenogenic mesenchyme of the masseters. The blue dashed lines circled the mandibular bones, while the white dotted boxes delineated masseter, which were magnified in the left-lower of each image. (T: Tongue; Ps: Palatal Shelves). **e**–**j** In situ hybridization showed the tenogenic markers and extracellular matrix gene expression in the E14.5 WT and *Osr2-cre; Rosa26R-Fgf8* masseter tendons. The *Scx* expression in the masseter tendons of the E14.5 WT (**e**) and *Osr2-cre; Rosa26R-Fgf8* mice (**f**). The *Tenomodulin* expression in the masseter tendons of the E14.5 WT (**g**) and *Osr2-cre; Rosa26R-Fgf8* mice (**h**). The blue dotted lines in **e**–**h** circled the mandibular bones. The *Tenascin C* expression in the masseter tendons of the E14.5 WT (**i**) and *Osr2-cre; Rosa26R-Fgf8* mice (**j**). The black arrowheads in **e**, **g** and **i** pointed to the masseter tendons in E14.5 WT mice, while the red arrowheads in **f**, **h** and **j** pointed to the masseter tendons in E14.5 *Osr2-cre; Rosa26R-Fgf8* mice. (Scale bars: 200 μm). **k**–**n** The Masson staining and immunohistochemical staining with antibody against Myosin in the WT and *Osr2-cre; Rosa26R-Fgf8* mandibles. **k**, **l** The Masson staining showed the morphology of the tendon and myofibers in the E13.5 WT (**k**) and *Osr2-cre; Rosa26R-Fgf8* masseters (**l**). The images in the left-lower of **k** and **l** showed the Myosin immunostaining of the masseters in the dashed boxes of **k** and **l**, respectively. **m**, **n** The Masson staining showed the morphology of the tendon and myofibers in the E16.5 WT (**m**) and *Osr2-cre; Rosa26R-Fgf8* masseters (**n**). The images in the left-lower of **m** and **n** showed the Myosin immunostaining of the masseters in the dashed boxes of **m** and **n**, respectively. Scale bars: 200 μm

Since *Osr2-cre* activated *Rosa26R-Fgf8* allele in the tenogenic mesenchyme of masseter, we examined the development of *Osr2-cre;Rosa26R-Fgf8* masseter tendon. In the mandible of E13.5 *Osr2-cre;Rosa26R-mT/mG* mice, the tenogenic mesenchyme was condensing into the presumptive deep and superficial masseter tendons (Fig. 3a, a’), while in E13.5 *Osr2-cre; Rosa26R-Fgf8; Rosa26R-mT/mG* mandible (Fig. 3b, b’), the *Osr2-cre* positive mesenchyme at both the deep and superficial masseter tendons was still loose and obviously expended to subcutaneous tissue. At E15.5, the *Osr2-cre* positive mesenchyme in *Osr2-cre;Rosa26R-mT/mG* mandible had condensed into the deep and superficial masseter tendons (Fig. 3c, c’). In contrast, the *Osr2-cre* positive mesenchyme in E15.5 *Osr2-cre; Rosa26R-Fgf8; Rosa26R-mT/mG* mandible formed the loose fibrous tissues at the level of deep masseter tendon (Fig. 3d), and was sparsely distributed in the subcutaneous tissue at the level of the superficial masseter tendons (Fig. 3d’).

The enlarged and sparse fibrous tissues formed by *Osr2-cre* positive mesenchyme in *Osr2-cre;Rosa26R-Fgf8;Rosa26R-mT/mG* mandible implicated the impaired tenogenesis of masseter tendons. In situ hybridization found that the makers for tenogenic differentiation, *Scx* and *Tenomodulin* (*Tnmd*), and the extracellular matrix expressed in tendon, *Tenascin C* (*TnC*) were all robustly expressed in the masseter tendon of E14.5 WT mice (Fig. 3e, g, i). In contrast, the transcription of *Scx* and *Tnmd* in the E14.5 *Osr2-cre;Rosa26R-Fgf8* deep masseter tendon was remarkably weaker than those in the WT control (Fig. 3f, h), though *TnC* transcription was comparable to that in WT control (Fig. 3j). Moreover, compared to the separated domains in the WT masseter (Fig. 3e, g, i), the *Scx*, *Tnmd* and *TnC* domains in the *Osr2-cre;Rosa26R-Fgf8* mandible fused together (Fig. 3f, h, j). In the *Osr2-cre;Rosa26R-Fgf8;Rosa26R-mT/mG* mandible, the enlarged *Osr2-cre* positive mesenchyme for masseter tendon was close to mandibular bone (Fig. 3b, b’), while the *Scx*, *Tnmd* and *TnC* expressing domains in the *Osr2-cre;Rosa26R-Fgf8* mandible were separated from the mandibular bone by atypical tissue (Fig. 3f, h, j). All these results suggested that both the patterning and differentiation of the *Osr2-cre;Rosa26R-Fgf8* masseter tendons were disrupted.

Although *Osr2-cre* was not activated in masseter, Masson staining showed that the E13.5 *Osr2-cre;Rosa26R-Fgf8* masseter lacked the condensed tendon and fibrous myofibers as E13.5 WT masseter did (Fig. 3k, l). The myosin immunostaining showed the area and myofiber density of the *Osr2-cre;Rosa26R-Fgf8* masseter, especially in the deep portion (Fig. 3l), were much less than those of WT controls (Fig. 3k). Compared to the E16.5 WT masseters (Fig. 3m), the decreasing areas and myofibers became more evident in the *Osr2-cre;Rosa26R-Fgf8* masseter, especially in the deep portion (Fig. 3n), suggesting a regression in masseter resulting from the impaired tenogenesis. In addition, consistent to the *Scx*, *Tnmd* and *TnC* expressing domains which were separated from the *Osr2-cre;Rosa26R-Fgf8* mandibular bone (Fig. 3f, h, j), Masson staining also confirmed that the enlarged tenogenic mesenchyme separating *Osr2-cre;Rosa26R-Fgf8* masseter and mandibular bone was composed of the irregular loose and dense tissues (Fig. 3n), which differed the dense regular tendon in WT masseter (Fig. 3m).

### Altered cell proliferation in the mandibular bone and masseter tendon of *Osr2-cre;Rosa26R-Fgf8* mice

**Fig. 4:**
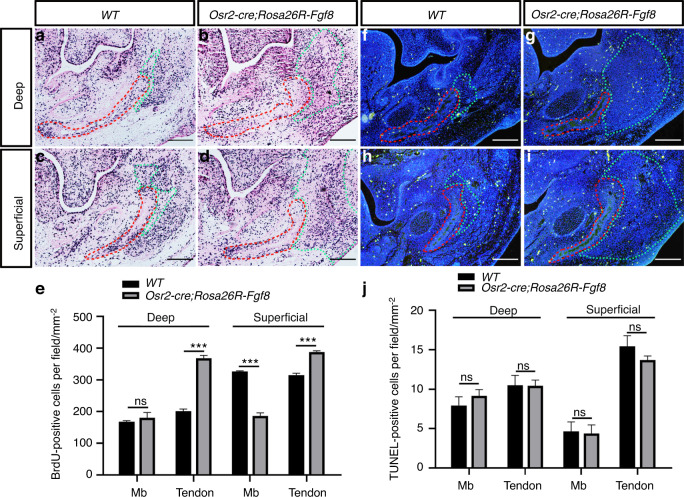
BrdU labeling and TUNEL assay in the osteogenic and tenogenic compartments of E13.5 *Osr2-cre; Rosa26R-Fgf8* mandibles. **a**–**d** The BrdU labeling assay showed the deep (**a**) and superficial masseter levels (**c**) of E13.5 WT mandibles, as well as the deep (**b**) and superficial masseter levels (**d**) of E13.5 *Osr2-cre; Rosa26R-Fgf8* mandibles. **e** Statistical assay indicated that at the deep masseter level, the densities of BrdU positive cells had no difference between E13.5 WT and *Osr2-cre;Rosa26R-Fgf8* mandibular bones (WT: 164.3 ± 3.3 per mm^2^ vs. *Osr2-cre; Rosa26R-Fgf8*: 184.0 ± 24.1 per mm^2^, *P* > 0.05); while the density of BrdU positive in the WT tenogenic compartments (198.3 ± 3.3 per mm^2^) was noticeably lower than that in *Osr2-cre;Rosa26R-Fgf8* tenogenic compartments (375.0 ± 16.7 per mm^2^, *P* < 0.001). In the superficial masseter level, the density of BrdU positive cells in osteogenic compartments of the E13.5 WT control were significantly higher than those of *Osr2-cre;Rosa26R-Fgf8* mandibles (WT: 329.7 ± 13.85 per mm^2^ vs. *Osr2-cre; Rosa26R-Fgf8*: 182.0 ± 13.9 per mm^2^, *P* < 0.001); in contrast, the density of BrdU positive cells in the tenogenic compartment of WT controls was greatly lower compared to *Osr2-cre;Rosa26R-Fgf8* mandibles (WT: 323.4 ± 5.47 per mm^2^ vs. *Osr2-cre; Rosa26R-Fgf8*: 386.5 ± 6.53 per mm^2^, *P* < 0.001). **f**–**i** The TUNEL assay showed the deep (**f**) and superficial masseter levels (**h**) of E13.5 WT mandibles, as well as the deep (**g**) and superficial masseter levels (**i**) of E13.5 *Osr2-cre; Rosa26R-Fgf8* mandibles. **j** Statistical assay indicated that in the osteogenic compartments, the densities of TUNEL positive cells at the levels of both deep and superficial masseter displayed little difference between E13.5 WT and *Osr2-cre;Rosa26R-Fgf8* mandibles (Deep: WT: 7.35 ± 0.94 per mm^2^ vs. *Osr2-cre; Rosa26R-Fgf8*: 8.66 ± 2.64 per mm^2^, *P* > 0.05; superficial: WT: 4.33 ± 1.28 per mm^2^ vs. *Osr2-cre; Rosa26R-Fgf8*: 3.67 ± 1.25 per mm^2^, *P* > 0.05); similarly, in the tenogenic compartments, the densities of TUNEL positive cells also showed no difference in deep and superficial masseter levels between the E13.5 WT and *Osr2-cre;Rosa26R-Fgf8* mandibles (Deep: WT: 11.67 ± 2.65 per mm^2^ vs. *Osr2-cre;Rosa26R-Fgf8*: 10.67 ± 1.70 per mm^2^, *P* > 0.05; superficial: WT: 16.33 ± 3.10 per mm^2^ vs. *Osr2-cre;Rosa26R-Fgf8*: 14.12 ± 3.65 per mm^2^, *P* > 0.05). The red dashed lines encircled the osteogenic compartments in mandibles, while the green gashed lines encircled teongenic compartments. Mb mandibular bone. Scale bars: 200 μm

To further address the changes in the tenogenic and osteogenic components of *Osr2-cre;Rosa26R-Fgf8* mandible, cell proliferation and survival were evaluated. BrdU labeling assay indicated that in the osteogenic compartments of the E13.5 *Osr2-cre;Rosa26R-Fgf8* mandible, the density of proliferating cells was comparable to WT controls at the level of deep masseter (Fig. 4a, b), but remarkably reduced at the level of superficial masseter (Fig. 4c, d). In contrast, the density of proliferating cells in the E13.5 *Osr2-cre;Rosa26R-Fgf8* tenogenic compartments were significantly increased at both levels of the deep and superficial masseter tendons (Fig. 4a–e). On the other hand, TUNEL assay showed that neither the osteogenic nor the tenogenic compartment of the E13.5 *Osr2-cre;Rosa26R-Fgf8* mandible displayed a discrepancy in the densities of apoptotic cells from the WT counterparts (Fig. 4f–j). These results suggested that the over-expressed *Fgf8* stimulated cell proliferation in the tenogenic mesenchyme, which led to the expanded *Osr2*-expressing domains, while suppressed cell proliferation in the *Osr2-cre;Rosa26R-Fgf8* mandibular bone.

### The tenogenic progenitors was converted into chondrogenic fate by ectopically activated *FGF8*

**Fig. 5:**
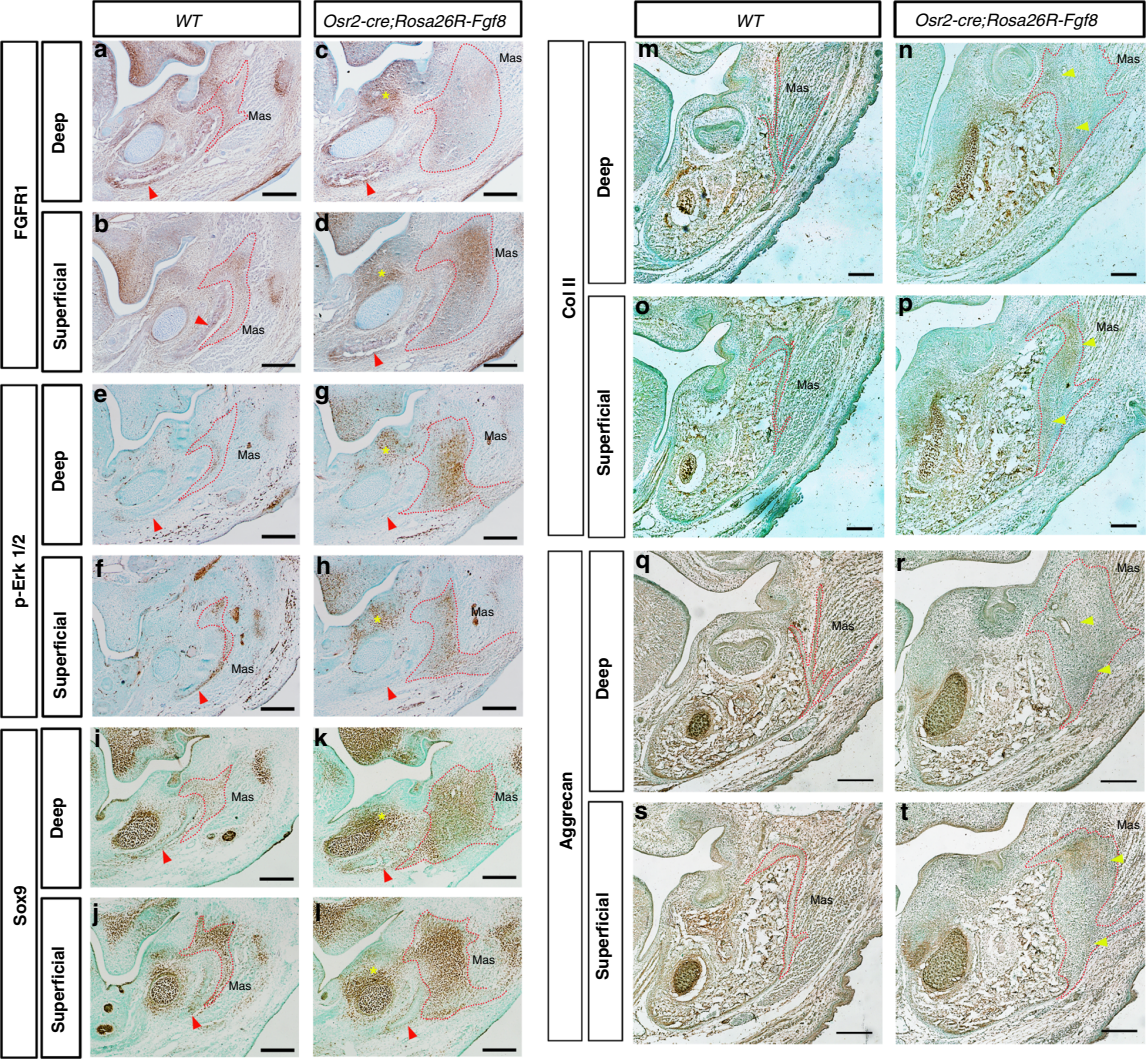
The immunostaining of FGF signaling and chondrogenic markers in *Osr2-cre; Rosa26R-Fgf8* mandibles. **a**–**d** The immunostaining of Fgfr1 in the cross sections of E13.5 WT deep (**a**) and superficial masseter levels (**b**), and the *Osr2-cre; Rosa26R-Fgf8* deep (**c**) and superficial masseter levels (**d**). **e**–**h** The immunostaining of p-Erk1/2 in the cross sections of E13.5 WT deep (**e**) and superficial masseter levels (**f**), and the *Osr2-cre; Rosa26R-Fgf8* deep (**g**) and superficial masseter levels (**h**). **i**–**l** The immunostaining of Sox9 in the cross sections of E13.5 WT deep (**i**) and superficial masseter levels (**j**), and the *Osr2-cre;Rosa26R-Fgf8* deep (**k**) and superficial masseter levels (**l**). **m**–**p** The immunostaining of Col II in the cross sections of E16.5 WT deep (**m**) and superficial masseter levels (**o**), and the *Osr2-cre; Rosa26R-Fgf8* deep (**n**) and superficial masseter levels (**p**). **q**–**t** The immunostaining of Aggrecan in the cross sections of E16.5 WT deep (**q**) and superficial masseter levels (**s**), and the *Osr2-cre; Rosa26R-Fgf8* deep (**r**) and superficial masseter levels (**t**). The red dotted lines encircled the masseter tenogenic mesenchyme; the red arrowheads in **a**–**l** indicated the positive signals in periosteum of mandibular bones, while the yellow arrowheads pointed to the staining of chondrogenic markers in masseter tendons; the yellow asterisks indicated the positive signals in the mesenchyme adjacent to Meckel’s cartile; Scale bars: 200 μm

To further explore the impact of ectopically activated *Fgf8* on masseter tendon, the major receptor for Fgf8, Fgfr1, was first examined in the E13.5 *Osr2-cre; Rosa26R-Fgf8* mandible. Immunostaining showed that in E13.5 WT mandible, Fribroblast Growth Factor Receptor 1 (Fgfr1) was localized at the buccal side of molar mesenchyme, the periosteal mesenchyme of mandibular bone, the perichondrial mesenchyme of Meckel’s cartilage, and the tenogenic mesenchyme of both deep and superficial masseters (Fig. 5a, b). While in E13.5 *Osr2-cre; Rosa26R-Fgf8* mandible, although the Fgfr1 expression in the periosteal and perichondrial mesenchyme was altered a little bit (Fig. 5c, d), the Fgfr1-expressing domains in the buccal molar mesenchyme and the tenogenic mesenchyme were expanded remarkably, especially in the superficial masseter level (Fig. 5c, d). Compared to WT controls (Fig. 5e, f), the phosphorylated- Extracellular Signal-Regulated Kinase 1/2 (p-ERK1/2) positive area was also increased in the E13.5 *Osr2-cre;Rosa26R-Fgf8* tenogenic and buccal molar mesenchyme (Fig. 5g, h). Notably, p-ERK1/2 staining which was obvious in the periosteal mesenchyme of WT mandibular bone (Fig. 5e, f) was diminished in the E13.5 *Osr2-cre;Rosa26R-Fgf8* counterpart (Fig. 5g, h). Sox9, the marker of osteogenic/chondrogenic progenitors, was activated in the Meckel’s cartilage, and the periosteal and tenogenic mesenchyme of E13.5 WT mandible (Fig. 5i, j). In the E13.5 *Osr2-cre;Rosa26R-Fgf8* mandible, Sox9-expressing domain was enlarged in the tenogenic mesenchyme and the mesenchyme surrounding Meckel’s cartilage, but changed little in the mandibular periosteal mesenchyme (Fig. 5k, l). These results implicated that the tenogenic mesenchyme in *Osr2-cre;Rosa26R-Fgf8* mandible was converted into chondrogenic fate. Although the chondrogenic extracellualr matrix, *Col2a1*, was not detected the E13.5 *Osr2-cre;Rosa26R-Fgf8* masseter tendon (data not shown). The collagen type II expression, which was constricted to the Meckel’s cartilage in E16.5 WT mandible (Fig. 5m, o), was ectopically activated in the tenogenic mesenchyme of *Osr2-cre; Rosa26R-Fgf8* masseter (Fig. 5n, p). Similarly, the marker for chondrogenic maturation, Aggrecan, which was only activated in the E16.5 WT Meckel’s cartilage (Fig. 5q, s), was also found ectopically activated in the tenogenic mesenchyme of E16.5 *Osr2-cre; Rosa26R-Fgf8* masseter (Fig. 5r, t). Interestingly, the ectopic collagen type II was found mainly in the enthesis side of *Osr2-cre; Rosa26R-Fgf8* tenogenic mesenchyme, while Aggrecan in the myotendious side. All these results indicate the conversion of the tenogenic mesenchyme into chondrogenic fate by the ectopically activated *Fgf8*.

### Constitutive activation of *Fgf8* in masseter does not affects the mandibular length

**Fig. 6:**
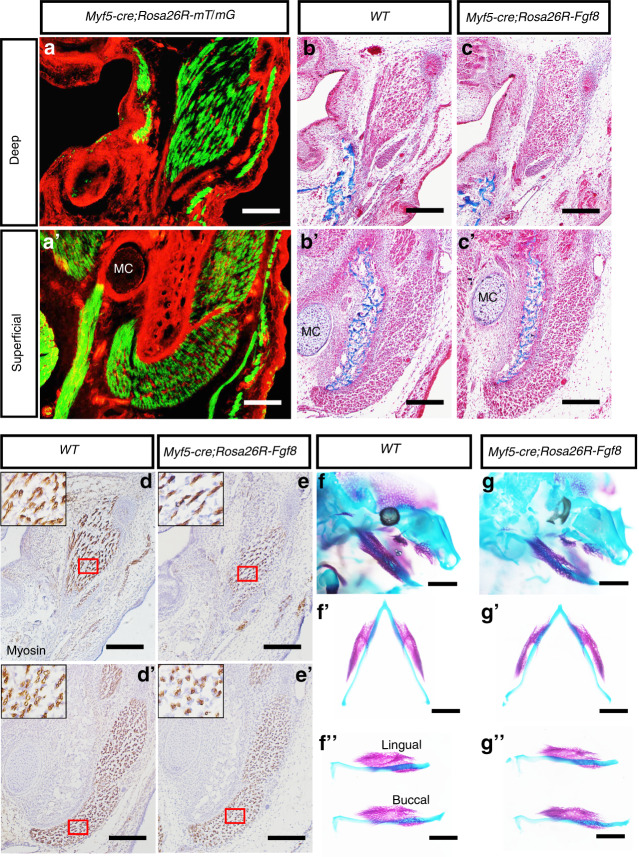
The developing masseter and madibular bone in *Myf5-cre; Rosa26R-Fgf8* mice. **a**, **a’** The cross views of the E16.5 *Myf5-cre;Rosa26R-mT/mG* deep (**a**) and superficial masseter levels (**a’**). **b, b’** The Masson staining of E15.5 WT deep (**b**) and superficial masseters (**b’**). **c**, **c’** The Masson staining of the E15.5 *Myf5-cre; Rosa26R-Fgf8* deep (**c**) and superficial masseters (**c’**). **d**, **d’** The Myosin staining of E15.5 WT deep (**d**) and superficial masseters (**d’**). **e**, **e’** The Myosin staining of the E15.5 *Myf5-cre; Rosa26R-Fgf8* deep (**e**) and superficial masseters (**e’**). The black solid boxes in the left-upper of the **d**, **d’**, **e** and **e’** were the magnified images of the red boxes in **d**, **d’**, **e** and **e’**, respectively. **f**, **f’**, **f”** The bone and cartilage staining showed the gross craniofacial view (**f**), and the ventral (**f’**) and lateral views (**f”**) of the mandibular bone in E15.5 WT mice. **g**, **g’**, **g”** The bone and cartilage staining showed the gross craniofacial view (**g**), and the ventral (**g’**) and lateral views (**g”**) of the mandibular bone in E15.5 *Myf5-cre;Rosa26R-Fgf8* mice. MC Meckel’s Cartilage, Scale bars in **a**–**e** and **a’**–**e’**: 200 μm; Scale bars in **f**, **g**, **f’**, **g’**, **f”** and **g”**: 2 mm

Since the constitutive activation of *Rosa26R-Fgf8* allele by *Wnt1-cre* suppressed myogenesis,^42^ the regression of *Osr2-cre;Rosa26R-Fgf8* masseter may result from the Fgf8 secreted from *Osr2-cre* positive cells. Thus, we activated *Rosa26R-Fgf8* allele by *Myf5-cre* to examine the effect of Fgf8 on myogenesis. The E16.5 *Myf5-cre; Rosa26R-mT/mG* mice displayed the Cre activity confined to the muscular components in mandible, such as the deep and superficial masseter, mylohyoideus, buccinator and even the subcutaneous muscles (Fig. 6a, a’). At E15.5, Masson staining showed that the *Myf5-cre;Rosa26R-Fgf8* myofibers and tendons of the deep and superficial masseters were comparable to WT control (Fig. 6b, b’, c, c’). Immunostaining of Myosin showed that although the densities of the masseter myofibers of the deep (Fig. 6d, e) and superficial masseters (Fig. 6d’, e’) had no discrepancy between E15.5 WT and *Myf5-cre;Rosa26R-Fgf8* mice, the intensity of Mysoin staining in *Myf5-cre;Rosa26R-Fgf8* masseter (Fig. 6e, e’) was a little slighter than that in WT control (Fig. 6d, d’), implying the suppressed maturation of masseter myofibers by Fgf8. Even though, both the Meckel’s cartilage and the osteogenic components of E15.5 *Myf5-cre;Rosa26R-Fgf8* mandible (Fig. 6g, g’, g”) were comparable in length to the WT counterparts (Fig. 6f, f’, f”). Thus, the regression of *Osr2-cre;Rosa26R-Fgf8* masseter was not attributed to Fgf8 emanated from the tenogenic mesenchyme.

### Abrogating the tenogenic progenitors or myoblasts of masseter also results in micrognathia

**Fig. 7:**
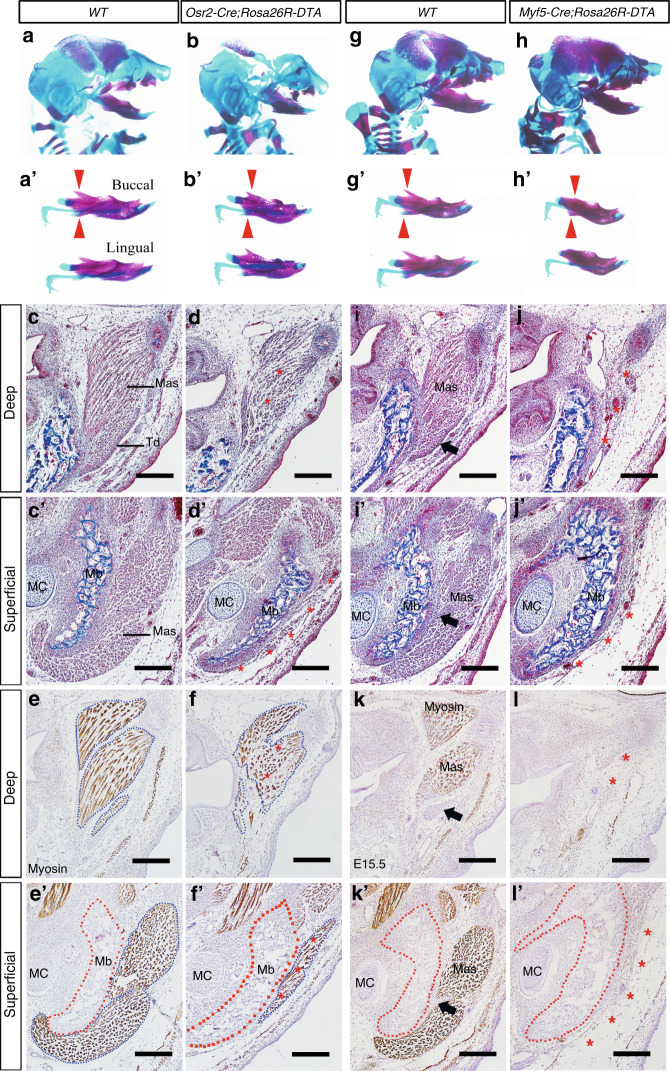
The skeleton and masseters in the mandibles of *Osr2-cre; Rosa26R-DTA* and *Myf5-cre; Rosa26R-DTA* mice. **a**, **a’** The gross craniofacial view (**a**) and the lateral view (**a’**) of E15.5 WT mandibular bone. **b**, **b’** The gross craniofacial view (**b**) and the lateral view (**b’**) of the E15.5 *Osr2-cre;Rosa26R-DTA* mandibular bone. (The red arrowheads in **a’** and **b’** pointed to the coronoid and angular processes). **c**, **c’** Masson staining showed the cross views of E15.5 WT deep (**c**) and superficial massters (**c’**). **d**, **d’** The cross views of E15.5 *Osr2-cre; Rosa26R-DTA* deep (**d**) and superficial masseter (**d’**). **e**, **e’** Myosin staining showed the E15.5 WT deep (**e**) and superficial masseter (**e’**). **f**, **f’** Myosin staining showed the E15.5 *Osr2-cre; Rosa26R-DTA* deep (**f**) and superficial masseter (**f’**). The blue dotted lines circled the masseter, while the red dotted lines circled mandibular bones. The red asterisks in **d**, **d’**, **f** and **f’** delineated the regressed myofibers of *Osr2-cre; Rosa26R-DTA* masseters. (Mas masseter, MC Meckel’s cartilage, Mb mandibular bone, Td masseter tendon. Scale bars: 200 μm). **g**, **g’** The gross craniofacial view (**g**) and the lateral view (**g’**) of E16.5 WT mandibular bone. **h**, **h’** The gross craniofacial view (**h**) and the lateral view (**h’**) of the E16.5 *Myf5-cre;Rosa26R-DTA* mandibular bone. (The red arrowheads in **g’** and **h’** pointed to the coronoid and angular processes). **i**, **i’** Masson staining showed the cross views of E16.5 WT deep (**i**) and superficial masseter (**i’**). **j**, **j’** The cross views of the E16.5 *Myf5-cre; Rosa26R-DTA* deep (**j**) and superficial masseter (**j’**). **k**, **k’** Myosin staining showed the E16.5 WT deep (**k**) and superficial masseter (**k’**). **l**, **l’** Myosin staining showed the E16.5 *Myf5-cre; Rosa26R-DTA* deep (**l**) and superficial masseter (**l’**). The red dotted boxes delineated mandibular bones. The black arrows in **i**, **i’**, **k** and **k’** indicated the tendons of the WT masseters, while the red asterisks in **j**, **j’**, **l** and **l’** delineated the diminished *Myf5-cre; Rosa26R-DTA* masseters. The red asterisks in **j**, **j’**, **l** and **l’** delineated the diminished *Myf5-cre; Rosa26R-DTA* masseters. Mas masseter, MC Meckel’s cartilage, Mb Mandibular bone, Td masseter tendon. Scale bars: 200 μm

To address whether the micrognathia of *Osr2-cre;Rosa26R-Fgf8* mice resulted from the disrupted development of masseter tendons, we exploited *Osr2-cre;Rosa26R-DTA* mice, in which the tenogenic progenitor of masester was eliminated, to check the influence of masseter tendon development on mandibular bone. Compared to the WT littermate (Fig. 7a, a’), E15.5 *Osr2-cre;Rosa26R-DTA* mice displayed reduced lengths in both the Meckel’s cartilage and the mandibular bone (Fig. 7b, b’). Similar to the micrognathia seen in *Osr2-cre;Rosa26R-Fgf8* mice, the shortened *Osr2-cre; Rosa26R-DTA* mandibular bone also lacked the well-developed coronoid and angular processes (Fig. 7b”). Histological sections indicated that compared to E15.5 WT masseter tendons (Fig. 7c, c’), the tenogenic components of the E15.5 *Osr2-cre;Rosa26R-DTA* deep and superficial masseters were completely lost (Fig. 7d, d’). Compared to WT deep and superficial masseters (Fig. 7e, e’), although the masseters was still found in the E15.5 *Osr2-cre; Rosa26R-DTA* mandible, the density, and length of masseter myofibers were significantly decreased, especially in the superficial masseter (Fig. 7f, f’). To further verify that both the well-developed tendon and masseter were essential for the normal mandibular bone, *Myf5-cre;Rosa26R-DTA* mice were exploited, in which all the myoblasts were abrogated. Similar to *Osr2-cre;Rosa26R-Fgf8* and *Osr2-cre;Rosa26R-DTA* mice, E16.5 *Myf5-cre; Rosa26R-DTA* mice exhibited the shorter Meckel’s cartilage and mandibular bone (Fig. 7h, h’), as well as the almost diminished coronoid, angular and condylar processes (Fig. 7h’) compared to the WT controls (Fig. 7g, g’). Both Masson staining and Myosin immunostaining indicated that in contrast to the clearly distinguished myofibers and tendons of E16.5 WT deep and superficial masseters (Fig. 7i, i’, k, k’), neither the myogenic nor tenogenic components could be found in the E16.5 *Myf5-cre; Rosa26R-DTA* massters (Fig. 7j, j’, l, l’).

### Impaired tenogenesis and myogenesis reduce mechanical loading and osteogenic specification in the mandibular bones

**Fig. 8:**
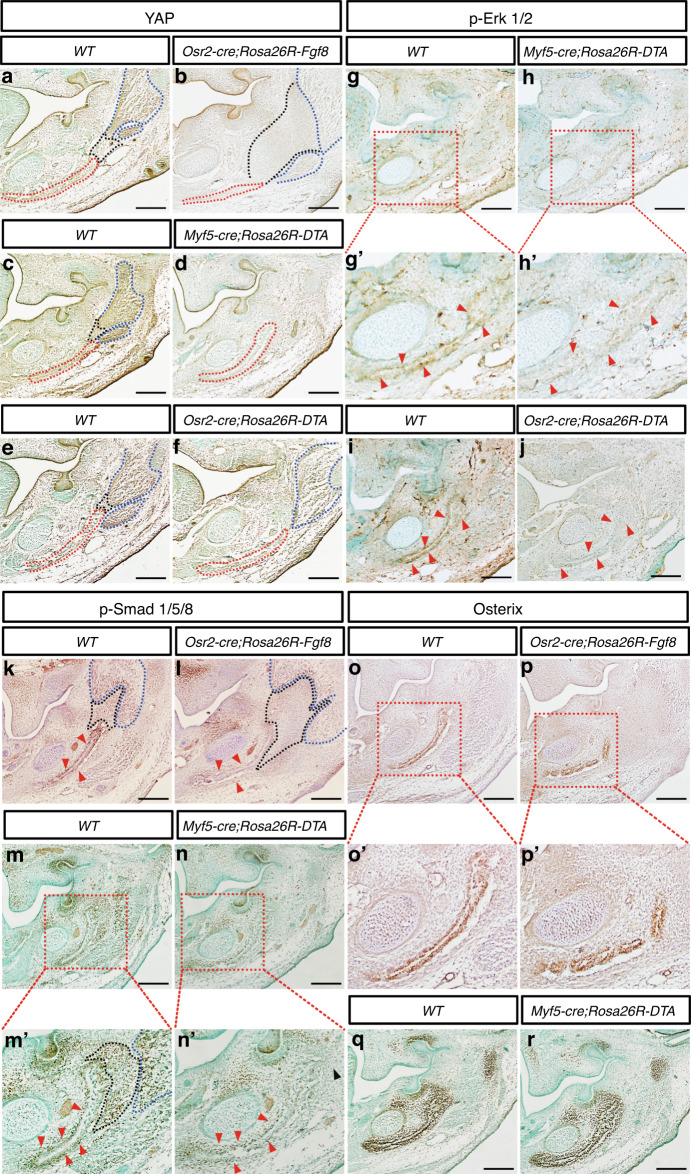
The markers of mechanical sensation and osteogenic differentiation in the mandibles with disabled tendons and masseters. **a**–**f** The immunostaining of Yap in E13.5 WT (**a**) and *Osr2-cre; Rosa26R-Fgf8* mandibles (**b**), E13.5 WT (**c**) and *Osr2-cre; Rosa26R-DTA* mandibles (**d**), as well as E13.5 WT (**e**) and *Myf5-cre; Rosa26R-DTA* mandibles (**f**). (**g**, **g’**, **h**, **h’**, **i**, **j**) The immunostaining of p-Erk1/2 in E13.5 WT (**g**) and *Myf5-cre; Rosa26R-DTA* mandibles (**h**), as well as E13.5 WT (**i**) and *Osr2-cre; Rosa26R-DTA* mandibles (**j**). The boxed areas in **g** and **h** were amplified in **g’** and **h’**, respectively. **k**, **l**, **m**, **m’**, **n**, **n’** The immunostaining of p-Smad1/5/8 in E13.5 WT (**k**) and *Osr2-cre; Rosa26R-Fgf8* mandibles (**l**), as well as E13.5 WT (**m**) and *Myf5-cre; Rosa26R-DTA* mandibles (**n**). The boxed areas in m and **n** were amplified in **m’** and **n’**, respectively. **o**, **o’**, **p**, **p’**, **q**, **r** The immunostaining of Osterix in E13.5 WT (**o**) and *Osr2-cre; Rosa26R-Fgf8* mandibles (**p**), as well as E13.5 WT (**q**) and *Myf5-cre; Rosa26R-DTA* mandibles (**r**). The boxed areas in **o** and **p** were amplified in **o’** and **p’**, respectively. Red dashed lines encircled masseters, blue dashed lines encircled mandibular bones, and the black dashed lines encircled masseter tendons; the red arrowheads pointed to the periosteal mesenchyme of mandibular bones; Scale bars: 200 μm

To explore how the degenerated tendons or masseters resulted in micrognathia, the mechanical sensory signaling, Hippo-Yes-Associated Protein (Yap) signaling, was examined in the E13.5 mice with defects in tendons or masseters. In the E13.5 WT mandibles, Yap was detected in both the developing mandibular bone and the masseter, but excluded from the masseter tendons (Fig. 8a, c, e). By contrast, the Yap expression was almost diminished in the E13.5 mandibles of *Osr2-cre;Rosa26R-Fgf8* (Fig. 8b) and *Myf5-cre; Rosa26R-DTA* mice (Fig. 8d), and was noticeably decreased in the E13.5 *Osr2-cre; Rosa26R-DTA* masseter and mandibular bone (Fig. 8f), which suggested the dramatic decrease in the mechanical loading on mandibular bone and masster because of the disabled masseters or tendons. Since Erk signaling was downregulated in the osteogenic mesenchyme of *Osr2-cre;Rosa26R-Fgf8* mandibular bone (Fig. 5g, h), *Myf5-cre; Rosa26R-DTA* and *Osr2-cre; Rosa26R-DTA* mice were exploited to address whether mechanical loading influences mandibular bone through Erk signaling. Compared to WT controls (Fig. 8g, g’, i), the immunostaining of p-Erk1/2 became remarkably weaker in the periosteal mesenchyme of E13.5 *Myf5-cre; Rosa26R-DTA* and *Osr2-cre; Rosa26R-DTA* mandibular bones (Fig. 8h, h’, j), implicating that mechanical force promotes mandibular osteogenesis through Yap-Erk signaling. Then, the influence of mechanical loading on the osteogenic differentiation in mandibular bone was assessed by the activities of BMP-Smad signaling and Osterix. The immunostaining of p-Smad1/5/8 was detected in E13.5 WT mandibular bone, tendon and masseter (Fig. 8k, m, m’). While in the E13.5 *Osr2-cre;Rosa26R-Fgf8* (Fig. 8l) and *Myf5-cre;Rosa26R-DTA* mandibles (Fig. 8n, n’), the p-Smad1/5/8 staining became noticeably fainter in mandibular bones, and even disappeared with the degenerated masseters and tendons. In contrast, Osterix staining in mandibular bones showed little difference between the E13.5 WT (Fig. 8o, o’, q) and *Osr2-cre;Rosa26R-Fgf8* (Fig. 8p, p’) or *Myf5-cre;Rosa26R-DTA* mice (Fig. 8r). Since BMP-Smad signaling is involved in both the specification of osteogenic progenitors and the differentiation of osteoblasts,^45^ while Osterix only contributes to osteoblastic differentiation, the reduced activity of BMP-Smad signaling in the mandibular bone implicated that the loss of mechanical force impaired osteogenic specification of mandibular bones, instead of the osteoblastic differentiation. This speculation was supported by microCT analysis on E18.5 *Osr2-cre;Rosa26R-Fgf8* mandibular bone (Supplementary Fig. 2), in which although the size and angular process were obviously smaller (Supplementary Fig. 2c), and even the lingual alveolar bone was absent (Supplementary Fig. 2d) compared to the WT control (Supplementary Fig. 2a,b), the indices of bone mass showed no difference from those of the controls (Supplementary Fig. 2e).

## Discussion

In this study, we investigated the causality between micrognathia and the impaired tenogenesis of masseter tendons, and how ectopic Fgf8 disrupted the development of masseter tendon. *Osr2-cre; Rosa26R-Fgf8* mouse embryos exhibited a typical PRS, which was characterized by micrognathia, undescended tongue, and cleft palate. Although the primary defects in *Osr2-cre;Rosa26R-Fgf8* palatal shelves have been described previously,^46^ the undescended tongue indeed contacted the palatal shelves and blocked their elevation, which recapitulates the process through which micrognathia initiates PRS. Intriguingly, as the initial factor of PRS, the micrognathia in *Osr2-cre;Rosa26R-Fgf8* mice resulted secondarily from the impaired tenogenesis of masseter tendons, which disrupted the osteogenic specification by reducing the mechanical force transmitted to mandibular bone. We further demonstrated that in *Osr2-cre;Rosa26R-Fgf8* mice, the conditional *Fgf8* knock-in allele was indeed ectopically activated by *Osr2-cre* in the presumptive mesenchyme for masseter tendon, and converted the tenogenic mesenchyme into chondrogenic fate, which reduced the mechanical force transmitted to mandibular bone. Consequently, the mechanical force generated by masseter contraction also became weakened, which eventually led to masester regression.

### *Osr2-cre* mice could be applied for studies of craniofacial tenogenesis

The *odd-skipped related* (*Osr*) gene family contains two members, *Osr1* and *Osr2*, both of which are zinc-finger transcription factors.^47^ According to the expression pattern and gene regulatory network, *Osr1* and *Osr2*, together with *Egr1*, *Kruppel like factor 2* (*Klf2*), and *Kruppel like factor 4* (*Klf4*) were believed to regulate the connective tissue subtype differentiation.^48^ In situ hybridization indicated that during limb development, the transcription of *Osr1* and *Osr2* was detected in the mesenchyme for presumptive synovial joints, but excluded from chondrogenic elements, which was coincided with the expression pattern of *Growth Differentiation Factor 5* (*Gdf5*). Although *Osr1* null mutant mice died of heart failure at mid-gestation, *Osr2*^*−/−*^ mice indeed showed the deformed and/or fused cartilages in synovial joints.^49^ Further detailed cell tracing experiments confirmed that *Osr1* and *Osr2-*expressing joint mesenchyme was overlapped in some extent with the muscle connective tissues and tendon progenitors marked by *Transcription Factor 4* (*Tcf4*) and *Scx*.^50^ Therefore, the *Cre* transgene driven by *Osr2* promoter could be activated in tenogenic mesenchyme.

In the developing murine craniofacial region, *Osr1* is only activated throughout the tongue mesenchyme from E12.5 on,^51^ while *Osr2* transcripts are detected in the lateral mesenchyme of palatal shelves, the dental mesenchyme (gradually extended from lingual to buccal side), the peripheral tongue mesenchyme,^52,53^ as well as the maxillary and mandibular mesenchyme immediately underneath oral epithelium. Hence, compared to the *Wnt1-cre* transgene which is activated at E9.5-10.5 and throughout the neural crest-derived craniofacial mesenchyme,^54^ the *Osr2-cre* mouse line has been applied in the study of palate and tooth development more and more widely.^27,55–57^ Actually, several cases of cleft palate resulting from the *Wnt1-cre-*driving conditional allele knockouts have been identified secondary to the micrognathia by deleting the same conditional alleles via *Osr2-cre*.^29,30,58^ In addition to the *Cre* expression confined to the palatal and dental mesenchyme, the *Osr2-cre* activated at E12.5 in the lateral mesenchyme connecting maxillary and mandibular processes has not been utilized in the study of craniofacial development. In this study, *Osr2-cre;Rosa26R-mT/mG* reporter mice demonstrated that the *Osr2-cre* positive mesenchyme connecting the maxillary and mandibular arches developed into the tendons of deep and superficial masseters. Moreover, our latest study showed that the tendons of the muscles in soft palate, including aponeurosis, were also *Osr2-cre* positive.^57^ Since *Scx-cre* mice usually exhibit a robust Cre activity after E13.5,^59^ the *Osr2-cre* mouse line is supposed to be an ideal tool for the study of craniofacial tenogenesis, especially for the early events in tenogenesis.

### Ectopically activated *Fgf8* converts the tenogenic progenitors into chondrogenic fate

Both *Fgf4* and *Fgf8* were only activated in the developing avian tendons, while the developing mammalian tendon was devoid of *Fgf8* expression.^40,41^ Since the tenogenic proliferation and differentiation were promoted only by *Fgf4*, the role of FGF8 in tenogenesis remians elusive.^41^ Our previous study showed that the constitutively activated *Fgf8* by *Wnt1-cre* suppressed the multi-lineage differentiation of the neural crest-derived craniofacial mesenchyme and maintained their stem cell status.^42^ In the present study, the ectopically activated *Fgf8* by *Osr2-cre* greatly promoted the proliferation of tenogenic mesenchyme, but disrupted both the patterning and the tenogenic differentiation of masseter tendon. Since the tenogenic progenitors are putatively derived from the Sox9^+^ cells on the surface of skeleton primordium,^60^ and the tenogenic fate of these progenitors is committed once *Sox9* expression was downregulated and *Scx* expression is up-regulated,^61,62^ the enhanced Sox9 expression in *Osr2-cre; Rosa26R-Fgf8* tenogenic mesenchyme implicates a conversion of the tenogenic fate into chondrogenic fate. This is supported by the ectopic expression of Collagen type II induced in the palatal mesenchyme of *Shox2-cre;Rosa26R-Fgf8* mice.^43^ However, the expression of Collagen type II was not detected until E16.5, along with the ectopically activated Aggrecan (an ECM for mature cartilage) and the absence of pre-hypertrophic chondrocytes, suggesting an atypical chondrogenic differentiation in the converted tenogenic mesenchyme of *Osr2-cre;Rosa26R-Fgf8* mandible. Furthermore, in the *Osr2-cre;Rosa26R-Fgf8* mandible, the activated *Fgf8* suppressed *Scx*, *Tnmd,* and *TnC* expression, but induced Fgfr1 expression and activated ERK signaling in the tenogenic mesenchyme. Previous study indicated that in the developing skeleto-muscular system, *Fgfr1* was specifically activated in tenogenic mesenchyme, *Fgfr2,* and *Fgfr3* in osteogenic/chondrogenic progenitors, and *Fgfr4* in myogenic compartments.^40^ A recent study reported a Sox9^+^/Scx^+^ population on the surface of mandibular bone as the bipotent progenitors for tenocytes or chondrocytes, however, inactivation of *Fgfr2* would decrease *Scx* expression in these progenitors and promote them to differentiate into chondrocytes.^63^ However, the expression pattern and intensity of FGFR2-4 showed no difference between WT and *Osr2-cre; Rosa26R-Fgf8* mandibles (data not shown). Thus, the tenogenic mesenchyme in craniofacial region was suggested to possess a competence for chondrogenic differentiation, and FGF8 converted the tenogenic mesenchyme into chondrogenic fate through Fgfr1-Erk1/2-Sox9 pathway.

### Disrupted development of masseter tendon leads to micrognathia by reducing the mechanical force from the masseter

In the developing mandibular arch, *Osr2-cre* is activated in the dental and tenogenic mesenchyme, but is excluded from the osteogenic and myogenic tissues. Since *Myf5-cre;Rosa26R-Fgf8* mice displayed comparable mandible and masseter to the normal controls, the regressed *Osr2-cre;Rosa26R-Fgf8* masseter was suggested to result secondarily from the impaired development of masseter tendon, instead of the FGF8 secreted by the tenogenic mesenchyme. Furthermore, when *Rosa26R-Fgf8* transgene was activated by *2.3Kb Col1a1-Cre* or *Dmp1-cre*, the mice showed no alteration in their skeletons (data not shown). These findings excluded the possibility that the FGF8 emanated from *Osr2-cre* positive cells directly inhibited the mandibular osteogenesis.

Latest study showed that deletion of *Tbx5* with *Osr2-cre* led to the mis-patterning of limb tendons and muscle hypoplasia,^50^ indicating that tenogenesis is essential for the primary myogenesis in limbs. Consistently, degenerated masseters was detected in *Osr2-cre;Rosa26R-DTA* mice with the abrogation of tenogenic component, and there was no discernable tendon in the mandibles of *Myf5-cre;Rosa26R-DTA* mice, verifying the mutual dependence between craniofacial tenogenic and myogenic tissues.

On the other hand, during the morphogenesis of long bone, the tenogenic tissues not only sculpture bone shape by transmitting mechanical loading generated by muscle contraction,^64^ but also carve the secondary structures on bone surface in a paracrine manner.^38^ The shortened mandibular bone with the miniature coronoid and angular processes in *Osr2-cre;Rosa26R-Fgf8*, *Osr2-cre;Rosa26R-DTA*, and *Myf5-cre; Rosa26R-DTA* mice suggest that the mechanical force from muscle contraction and paracrine factors from tenogenic tissues exerted on the mandibular bone are disrupted. The Yap signaling, which senses the intracellular mechanical force in developing bones and muscles,^65–67^ was significantly downregulated in the myogenic and osteogenic compartments of *Osr2-cre;Rosa26R-Fgf8*, *Osr2-cre; Rosa26R-DTA*, and *Myf5-cre;Rosa26R-DTA* mandibles, which is consistent with the degenerated masseter and reduced mechanical force. Previous studies indicated that mechanical force could promote cell proliferation through ERK-YAP signaling.^68,69^ The increasing shear force enhanced the nuclear location of active ERK, which translocated YAP into cell nucleus, and activated cell cycle genes. This established interpretation was supported by the decreased cell proliferation in masseter tendons, the reduced ERK and YAP intensity in the periosteal mesenchyme of mandibular bone of *Osr2-cre;Rosa26R-Fgf8* mice. It is worth of noticing that both the ERK and BMP-Smad signaling pathways were obviously downregulated in the periosteal mesenchyme of the *Osr2-cre;Rosa26R-Fgf8*, *Osr2-cre; Rosa26R-DTA* and *Myf5-cre; Rosa26R-DTA* mandibular bone, while the Osterix expression seemed unaffected. Since both the ERK and BMP-Smad signaling pathways are involved in the specification of osteogenic progenitors,^30,45^ these findings implicate that the loss of mechanical force in the developing mandibular bone represses the osteogenic specification of the mandibular mesenchyme, but does not impact the osteoblastic differentiation. This speculation was coincided with the microCT results, because the normal bone mass of E18.5 *Osr2-cre;Rosa26R-Fgf8* mandibular bone indicated an unffected osteoblatic differentiation and mineralization, while the reduced sizes of mandibular bone and angular process, as well as the lost lingual alveolar bone could be attributed to the decreased amount of osteoblast progenitors.

Previous study on Treacher Collins Syndrome demonstrated that the increased oxidative pressure resulting from genetic mutation led to the excessive apoptosis of neural epithelial cells and premigratory neural crest cells, which reduced the amount of progenitors for the presumptive mandibular arch.^9^ Since neural crest cells contributes various types of tissues,^70^ the miracognathia resulting from genetic mutation is usually companied with other defects, namely, the syndromic micrognathia. However, more than two thirds micrognathia are sporadic and non-syndromic without systemic defects. Our study showed that the disrupted tenogenesis in masseter tendon results in not only micrognathia, but also degenerated masseter, which provides a novel insight for the etiology of micrognathia.

## Materials and methods

### Mouse lines

The *Osr2-cre*,^71^ *Rosa26R-Fgf8*,^72^ *Myf5-cre* (Stock No. 007893), *Rosa26R-mT/mG* (Stock No. 007676), and *Rosa26R-DTA* (Stock No. 009669) mice have been described previously. Genotyping was carried out using PCR on tail tip DNA. All these mice were fed and maintained in the Specific Pathogenic Free System of the Institute of Genome Engineered Animal Models for Human Diseases at Dalian Medical University. To get timed pregnant mice, the female mice and male mice were mated in the 12 h light/12 h dark cycle. The morning in which vaginal plug was detected was recorded as Embryonic Day 0.5 (E0.5). All procedures followed the protocol approved by the Animal Care and Use Committee at Dalian Medical University (Protocol No. AEE20016).

### Cryostat section

The *Osr2-cre;Rosa26R-mT/mG* and *Myf5-cre;Rosa26R-mT/mG* embryos were fixed in the ice-cold mixture containing 4% paraformaldehyde and 15% sucrose over-night and then, in 30% sucrose solution for dehydration. The fixed samples were embedded with O.C.T. compound (Tissue-Tek, Sakura^®^Finetek, VWR, Torrance, CA, United States) for 10 µm serial cryostat sections in a cryostat microtome. The images were taken by the Olympus DP72 microscope (Olympus, Tokyo, Japan) immediately after sectioning.

### Bone and cartilage staining

The mouse embryos were fixed in absolute alcohol overnight after the skin and internal organs removed, and degreased in acetone for 2–4 days. Alizarin Red S (0.1% in 70% ethanol) was used for bone staining and Alcian Blue (0.3% in 70% ethanol) for cartilage staining. After 3–4 days staining in the Alizarin Red S and Alcian Blue mixture, the 25% glycerol solution containing 2% potassium hydroxide was used to remove the excess staining. Finally, the stained samples were stored in absolute glycerol.

### Histological section and Masson staining

The embryonic mouse heads were fixed in 4% paraformaldehyde in phosphate-buffered saline at 4 °C, paraffin embedding after ethanol gradient dehydration and clearing with xylene, and then, sectioned at 10 μm for Masson staining (Biebrich scarlet-acid fuchsin solution for cytoplasm, fibrin, and muscles, and Aniline blue for collagen fibers).

### In situ hybridization (ISH)

The embryonic mouse heads were fixed in 10% neutral-buffered formalin overnight at room temperature, ethanol-dehydrated, paraffin-embedded, and sectioned at 8 µm. The expression patterns of *Scleraxis (Scx)*, *Tenomodulin (Tnmd)*, *Tenascin (Tnc)* and *Col1a1* were examined by in situ hybridization using the RNAscope 2.5 Assay kit (Advanced Cell Diagnostics, Newark, CA, USA) on formalin-fixed paraffin sections following the manufacturer’s instructions.^73^ Hematoxylin was used for counter-staining.

### Immunohistochemistry

Histological sections were dewaxed in xylene and rehydrated with gradient alcohols. Antigen retrieval by boiling in citrate sodium buffer (pH 6.0) for 10 min. The sections were blocked in 3% H_2_O_2_ and methanol mixture for 20 min, and then, with 10% goat serum (Maixin Ltd., Fuzhou, China) and 0.3% Triton X-100 in PBS for 1 h at room temperature, and incubated overnight at 4 °C with the primary antibodies against p-Erk1/2 (Abcam, Catlog NO. Ab50011, in the dilution of 1:200), phosphorylated-Smad1/5/8 (p-Smad1/5/8, Cell Signaling Technology, Catlog NO. 13820S, in the dilution of 1:200), Sox9 (Abcam, Catlog NO. Ab185966, in the dilution of 1:1 000), Myosin (Zhongshan Golden Bridge, Catlog NO. ZM0196, in the dilution of 1:50), FGF Receptor 1 (Fgfr1, Cell Signaling Technology, Catlog NO. 9740S, in the dilution of 1:400), YAP (Cell Signaling Technology, Catlog NO. 14074T, in the dilution of 1:400), Collagen Type II (Col II, Proteintech, Catlog NO. 28459-1-AP, in the dilution of 1:800), Osterix (OSX, Abcam, Catlog NO. Ab209484, in the dilution of 1:100) and Aggrecan (Proteintech, Catlog NO. 13880-1-AP, in the dilution of 1:1 000), respectively. The horseradish peroxidase (HRP)-conjugated anti-rabbit/mouse IgG and 3,3′-diaminobenzidine (DAB) (Maixin Ltd., Fuzhou, China) were used as the secondary antibodies and color development at room temperature, respectively. Next, methyl green was used for counter-staining.

### BrdU (5-Bromo-2′-deoxyuridine) labeling and TUNEL (TdT-mediated dUTP nick end labeling) assay

To assess the cell proliferation in mandible and tendon, 10 mmol·L^−1^ BrdU was intraperitoneal injected (500 µL per 100 g body weight) to the timed-pregnant mice. After 30 min of injection, mice were sacrificed and embryos’ heads were fixed in Carnoy’s fixative for 4 h, ethanol-dehydrated, paraffin-embedded, and sectioned at 10 µm. Detection of BrdU-labeled cells in E13.5 was carried out using the Detection Kit II (Roche, Catlog NO. 11299964001). The sections were counter-stained with nuclear fast red. To assess the cell apoptosis, TUNEL assay was performed on 10 µm-thick paraffin sections with the In Situ Cell Death Detection Kit, POD (Roche, Catlog NO. 11684817910). The sections were counter-stained with DAPI. The density of the proliferating cells/apoptotic cells was calculated by the numbers of BrdU-positive/TUNEL-positive cells in defined area of mandible and tendon. Three independent BrdU-labeling and TUNEL operations were performed with three samples for statistical assay.

### Micro-computed tomography (microCT)

Mandibles of E18.5 WT and *Osr2-cre;Rosa26R-Fgf8* mice were scanned and reconstructed by a micro-computed tomography system (μCT35; Scanco Medical AG, Bassersdorf, Switzerland) with the current of 114 mA, the voltage of 70 kVp and the exposure time of 300 μs. 7 *Osr2-cre;Rosa26R-Fgf8* mice and their normal littermates from three litters were collected for the microCT scanning to evaluate the bone mass of mandibular bones.

### Statistical analysis

Experiments were performed at least three biological replicates for each group for statistical analysis. The mandible and tendon area were defined and estimated by Image J (version 1.46r, National Institutes of Health). In cell proliferation/TUNEL assay, the number of the BrdU/TUNEL labeled cell nuclei were also counted by Image J. Two-tailed unpaired Student’ *t* tests were applied for statistical analysis. Statistical result was present in mean values ± standard variations and the significance was defined as *P < 0.05, **P < 0.01 and ***P < 0.001.

## Supplementary information

Supplementary Figure 1

Supplementary Figure 2

Supplementary Figures legend

## Data Availability

The data of this study are available from the corresponding authors upon reasonable request.
