# Supplementary figures and images for: Disrupted tenogenesis in masseter as a potential cause of micrognathia
Source: Int J Oral Sci. 2022 Oct 18;14:50. doi: 10.1038/s41368-022-00196-y (PMC9579150; doi:10.1038/s41368-022-00196-y)

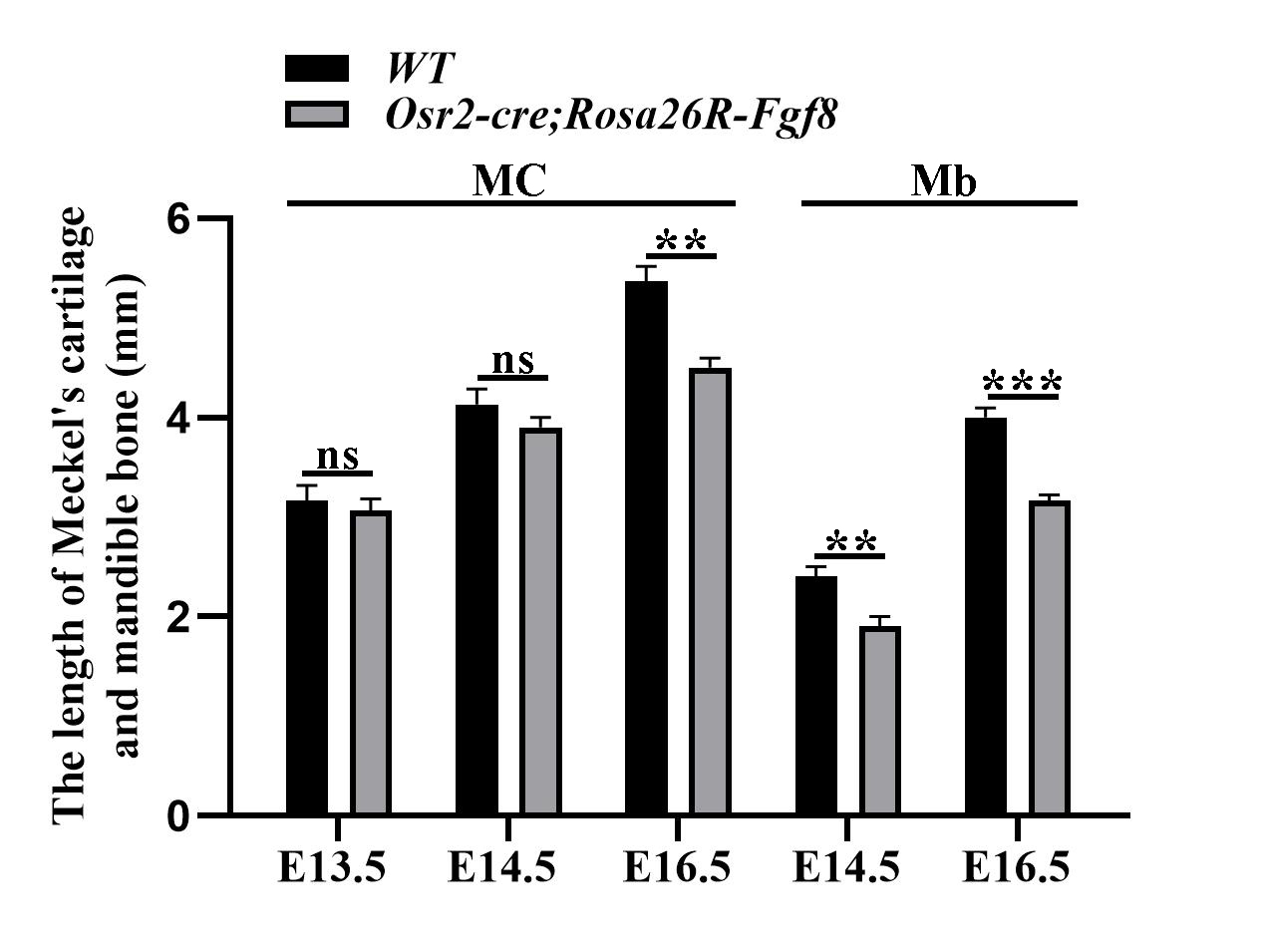

Supplement: Supplementary file 1 — Supplementary Figure 1 [file 41368_2022_196_MOESM1_ESM.jpg]

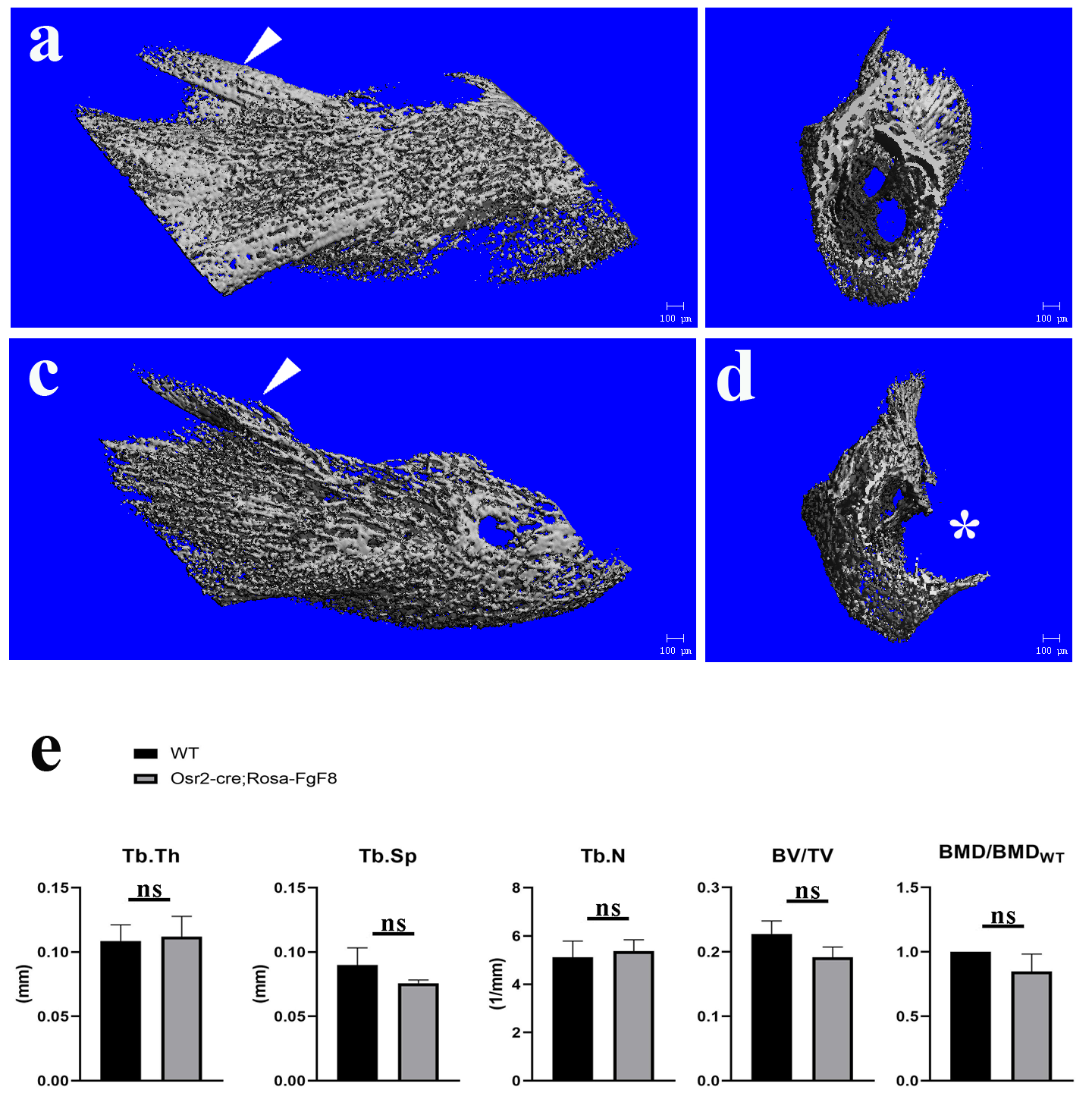

Supplement: Supplementary file 2 — Supplementary Figure 2 [file 41368_2022_196_MOESM2_ESM.tif]
