# Supplementary material for: Disrupted tenogenesis in masseter as a potential cause of micrognathia
Source: Int J Oral Sci. 2022 Oct 18;14:50. doi: 10.1038/s41368-022-00196-y (PMC9579150; doi:10.1038/s41368-022-00196-y)
Supplement: Supplementary file 3 — Supplementary Figures legend [file 41368_2022_196_MOESM3_ESM.docx]

**Supplementary Figure 1.** Statistical assay for the lengths of mandible bone and Meckel's cartilage. Statistically, the lengths of E13.5 WT Meckel’s cartilages (2.967±0.047 mm) showed no difference from those of E13.5 *Osr2-cre; Rosa26R-Fgf8* Meckel’s cartilages (2.867±0.447 mm; *p*=0.101); similarly, the E14.5 WT Meckel’s cartilages (4.133±0.124 mm) also had no difference in length from *Osr2-cre; Rosa26R-Fgf8* Meckel’s cartilages (3.905±0.081 mm; *p*=0.091); while the E16.5 *Osr2-cre; Rosa26R-Fgf8* Meckel’s cartilages (4.590±0.082 mm) were significantly shorter than WT controls (5.367±0.128 mm; *p*=0.0012). At least three WT and *Osr2-cre; Rosa26R-Fgf8* embryos from the same litters of each stage were collected for analysis. (ns: *p*>0.05; **: *p*<0.01;***: *p*<0.001)

In contrast to Meckel’s cartilage, the ossified mandibular bones in E14.5 WT mice (2.459±0.082 mm) were significantly longer than those of *Osr2-cre; Rosa26R-Fgf8* mice (1.922±0.081 mm; *p*=0.0036); at E16.5, the difference in the lengths of ossified mandibular bones between WT (4.122±0.017 mm) and *Osr2-cre; Rosa26R-Fgf8* mice (3.292±0.041 mm) became more significantly (*p*=0.00061)._At least three WT and *Osr2-cre; Rosa26R-Fgf8* embryos from the same litters of each stage were collected for analysis. (ns: *p*>0.05; **: *p*<0.01;***: *p*<0.001)

**Supplementary Figure 2.** MicroCT analysis on trabecular bone mass of E18.5 *Osr2-cre; Rosa26R-Fgf8* mandibular bones. **(a-d)** The lateral **(a)** and cross microCT images **(b)** of E18.5 WT mandibular bones, and the lateral **(c)** and cross microCT images **(d)** of E18.5 *Osr2-cre; Rosa26R-Fgf8* mandibular bones. White arrowheads pointed to angular processes, while the asterisk indicated a loss of lingual alveolar bone. **(e)** Statistical assay showed no significant difference in the trabecular thickness, space and number of the mandibular bones between E18.5 WT and *Osr2-cre; Rosa26R-Fgf8* mice. Consistently, there was also no discerpancy in the ratios of BV/TV and bone mineral densities between E18.5 WT and *Osr2-cre; Rosa26R-Fgf8* mandibular bones. (ns: *p*>0.05; scale bar is 100 μm)
